# A randomized controlled trial of a self-guided mobile app targeting repetitive negative thought to prevent depression in university students: study protocol of the Nurture-U Reducing Worry prevention trial

**DOI:** 10.1186/s12888-024-06079-2

**Published:** 2024-10-02

**Authors:** E. R Watkins., D. Phillips, T. Cranston, H. Choueiri, M. Newton, H. Cook, G. Taylor

**Affiliations:** 1 Sir Henry Wellcome Building for Mood Disorders Research, School of Psychology, University of Exeter, Exeter, EX4 4LN UK; 2 Clinical Trials Unit, University of Exeter Medical School, University of Exeter, Exeter, UK

**Keywords:** Depression, Well-being, University students, Mobile-health prevention, Randomised controlled trial, Worry, Rumination

## Abstract

**Background:**

Tackling poor mental health in university students has been identified as a priority in higher education. However, there are few evidence-based prevention initiatives designed for students. Repetitive Negative Thought (RNT, e.g. worry, rumination) is elevated in university students and is a well-established vulnerability factor for anxiety and depression. Furthermore, there are now evidence-based cognitive-behavioural interventions to tackle RNT. A mobile self-help cognitive-behavioural app targeting RNT, adapted for students may therefore be an effective, scalable, and acceptable way to improve prevention in students.

**Methods:**

An online single blind, two-arm parallel-group Randomised Controlled Trial (RCT) to examine the incidence of major depression and symptoms of anxiety and depression across 12 months in university students aged over 16 who screen into the study with self-reported high levels of worry and/or rumination and no current diagnosis of major depression. Eligible participants will be randomised to the active intervention arm (usual practice plus using a self-guided mobile app targeting RNT) or to the control arm (usual practice). In total, 648 participants aged over 16, with no current major depression, bipolar disorder or psychosis will be recruited from UK universities. Assessments will take place at baseline (pre-randomisation), 3 months and 12 months post- randomisation. Primary endpoint and outcome is incidence of major depression as determined by self-reported diagnostic criteria at 12-month follow-up. Depressive symptoms, anxiety, well-being, health-related quality of life, functioning and academic outcomes are secondary outcomes. Compliance, adverse events, and potentially mediating variables will be carefully monitored.

**Discussion:**

The trial aims to provide a better understanding of the causal role of tackling RNT (worry, rumination) using a self-help mobile app with respect to preventing depression in university students. This knowledge will be used to develop and disseminate innovative evidence-based, feasible, and effective mobile-health public health strategies for preventing common mental health problems.

**Trial registration:**

https://www.isrctn.com/ISRCTN86795807 Date of registration: 27 October 2022

## Background

University provides an opportunity for young people to develop independence, positive self-identity, good coping skills and social-emotional resources to stand them in good stead through their lives, as they face challenges like leaving home, meeting academic standards, making new friends, and managing finances. However, university is also a high-risk period for stress and poor mental health, which can result in increased drop-out, poorer academic outcomes, diminished employment opportunities and long-term disadvantage [1–3]. Mental health concerns are increasing amongst university students, with anxiety, depression, self-harm, and alcohol/substance abuse most common [4–8].

One of the major challenges for enhancing student mental health is the absence of proven effective prevention and wellbeing promotion initiatives [9, 10]*.* To tackle this key challenge, the current Nurture-U (NURTURE University) Prevention trial investigates the use of self-guided cognitive-behavioural strategies delivered by mobile app to prevent depression in university students. More specifically, this self-help intervention targets elevated repetitive negative thought (RNT) in the form of worry and rumination. RNT is a good intervention target to help students because it is a vulnerability factor that is robustly found to predict later depression, anxiety, alcohol misuse and eating disorders [11–14] and can be successfully reduced by cognitive-behavioural approaches [15–19], making it a tractable target for prevention. Furthermore, recent research has indicated that RNT is elevated in university students [5] and that it may be increasing over time in students [20], and that it specifically predicts poor mental health in students [13, 18, 19, 21]. Furthermore, RNT (e.g., worry) is a problem that students can easily self-identify and are motivated to seek help for, and that does not carry the potential stigma of more directly targeting anxiety and depression – targeting RNT may thus have the benefits recently proposed for indirect preventative interventions [22].

A version of cognitive-behavioural therapy (CBT) adapted to target RNT has been proven to be effective in reducing and preventing depression and anxiety in face-to-face therapy [15–17]. Moreover, a randomised controlled trial of a preventative intervention designed to target excessive levels of RNT in young people with elevated levels of worry and rumination found that both a group based and internet version of a rumination-focused Cognitive Behavioural Therapy programme (RF-CBT) significantly reduced RNT (*d* = 0.53 to 0.89) and halved the rates of caseness for self-reported major depression and generalized anxiety disorder over the next 12 months relative to a wait-list control [18]. A further trial involving 235 UK undergraduates with high-RNT found that a therapist-guided internet-based version of this RNT-targeting therapy reduced the risk of depression by 34% relative to controls over the next 15 months and that participants showed a significant improvement in RNT and depressive symptoms in the short to medium term [19]. A feasibility arm within this trial suggested that an unguided self-help version of this approach may prevent anxiety and depression in students [19] but this needs robust evaluation in a large-scale suitably powered proof-of-concept trial to test its potential as a highly scalable approach [23]*.*

Moreover, prior prevention work in students has focused on the use of internet-delivered interventions. However, it may be that the use of a mobile app has further advantages. First, the use of mobile phones is near ubiquitous in university students – in the UK within young people aged 16-24, in 2022, 98% had a smart phone [24]. Mobile apps can thus provide a highly scalable intervention, allowing very good coverage and reach and that is widely accessible and acceptable, and convenient that can be used anytime and anywhere, all of which are critical for an effective preventive intervention [25]. Second, an unguided self-help app would be non-consumable and can be used by a nearly unlimited number of people simultaneously unlike guided interventions, which are limited by the capacity of the coaches or therapists [26]. Third, mobile apps can help to integrate behavioural changes into daily life: the app is always on hand via the smartphone, making it well-suited for changing mental habits such as worry and rumination [27].

Despite a huge increase in the number of mobile-based mental health apps over the last ten years [28] only a small number have been developed with scientific rigour. Further, many do not utilise established treatment principles nor have they been rigorously tested in robust well-powered Randomised Controlled Trials (RCTs) [28–32]. While emerging evidence suggests that mobile-based applications can deliver efficacious treatment interventions for anxiety and depression [33, 34], there have not been many trials examining their use for prevention of poor mental health in young people and in university students particularly [28, 32]. Many studies have been under-powered, with less than 100 participants per trial arm.

The app in this study will adapt an existing proven intervention that targets a shift away from maladaptive worry and rumination, as well-established risk factors for poor mental health [11–14] to more adaptive problem-solving. This self-help intervention builds on proven cognitive-behavioural therapy principles and includes identifying warning signs for worry, repeated practice to train out of unhelpful habits and build helpful habits, and the training of useful alternative strategies such as being more specific, relaxation, problem-solving and self-compassion [15–19, 35]. The comparator condition will be usual practice, reflecting the standard experience of students not receiving the app, providing a pragmatic real-world control.

## Objective

The primary objective of this trial is to examine the efficacy of unguided digital RNT-targeting self-help relative to usual practice to prevent incidence of major depression over a 12-month period in university students with elevated risk as indexed by high levels of RNT. Secondary objectives are to examine the efficacy of this self-help app on secondary outcomes concerning mental well-being and mental health and associated costs, the maintenance of these effects at 12-month follow-up and to examine potential mediators and moderators of the beneficial effects (if any) of this intervention.

It is hypothesised that for university students with elevated worry and rumination, a self-guided mobile app that targets RNT added to usual practice will outperform usual practice in reducing incidence of depression across 12 months and reducing symptoms of worry, rumination, depression, and anxiety at 3 and 12 months.

## Methods

The study will be conducted and reported according to Consolidated Standards of Reporting Trials (CONSORT) [36, 37] and extensions for non-pharmacologic treatment interventions and multi-arm parallel-group randomised trials and CONSORT-EHEALTH for improving and standardising evaluation reports of Web-based and mobile health interventions [38].

### Study design

The trial design is a superiority parallel 2-arm individual-level single-blind Randomized Controlled Trial (RCT). All participants will be randomized into either receiving the self-help unguided self-help app incorporating CBT strategies targeting RNT in addition to usual practice or to receiving usual practice alone.

Potential participants for the trial provide initial consent to complete screening measures to determine if they are eligible to participate in the trial, i.e., showing elevated levels of RNT. Any potential participants who are found not to be eligible are automatically signposted to other sources of support. Once trial eligibility has been determined and consent to participate in the trial has been obtained, participants are individually selected at random (in a 1:1 ratio) to be offered the unguided self-help app or to continue with usual practice only.

### Recruitment and study settings

We seek to recruit 648 participants from within United Kingdom universities (focused on the partner universities in the grant: Exeter, Newcastle, Oxford, Cardiff, Southampton, and King’s College London, but also open to other UK universities). The recruitment strategy will include on-campus presentations, posters, stands, and presentations in lectures, online and website advertising; email to mailing lists; newsletters and other circulars and noticeboards within willing universities in the UK. A social media campaign will be designed and prepared to be carried out on different social networks (e.g., Facebook, Instagram, TikTok), including advertisements in social media.

### Eligibility criteria

Eligible participants will be: (1) over 16 years of age; (2) studying at a UK university; (3) able to provide informed consent; (4) having regular access to a smart phone (android or iOS), tablet, PC or laptop, necessary to use intervention; (5) available for the full duration of the trial (12 months); (6) able to complete consent and online questionnaires based on a basic literacy in English; (7) scoring above previously established cut-offs on standardized self-report measures of worry (Penn State Worry Questionnaire- Short-form, PSWQ-S) [39] and rumination (Ruminative Response Scale-Brooding, RRS-B) [40] , defined as scoring in the worst performing quarter on at least one of these measures, and scoring in the worst performing third on the second measure (in practice, this means scores of >11 for worst tercile, > 12 for worst quartile on the RRS-brooding scale and >24 for top tercile and >26 for top quartile on the Penn State Worry Questionnaire short-form). These thresholds are based on prior studies finding that individuals scoring in the worst quarter on measures of RNT have elevated risk for subsequent anxiety and depression [18, 19, 41].

Since this is a prevention trial, potential participants will be excluded at baseline if currently meeting diagnostic criteria for major depression (according to psychiatric DSM-V criteria), determined in structured self-report electronic screening using the LIDAS instrument [42]. Participants will also be excluded at baseline if presenting with highly elevated symptoms of depression indicating they require more specialist treatment. This is defined as having a score of 20 or higher on the Patient Health Questionnaire (PHQ-9) [43]. Other exclusion criteria include: active suicidality; any self-reported history of severe mental health problems such as bipolar disorder and psychosis and drug/alcohol dependence; and currently receiving psychological therapy, counselling or psychiatric medication including antidepressants.

### Screening and consent procedure

Potential participants who are interested in the study will be directed to our study website, (www.nurtureuniversity.co.uk) which provides further information. Interested participants can proceed directly to the Electronic Data Capture (EDC) system and undertake a brief pre-screener to check age and that the participant is a university student. If appropriate, the website visitor can watch a video version of the screening Participant Information Sheet (PIS) and/or read an electronic version of the PIS and an initial consent screen to provide contact details (email; mobile phone number), and to provide informed consent (screening consent form) to complete the screening questionnaires. After completing pre-screening, potential participants will be automatically emailed a copy of the screening PIS, privacy policy and completed screening consent form. Once this initial consent is provided, the participant will complete the screening assessment consisting of the LIDAS, PSWQ-S, RRS-B and PHQ-9 and if meeting eligibility criteria will be assigned to the trial (the screening process will in parallel be assessing participants for eligibility for a trial of internet cognitive behavioural therapy for students with elevated anxiety and depression).

Those meeting eligibility criteria following the screening assessment will then be asked to consent to take part in the trial after viewing a video version of the trial PIS and/or reading an electronic version of the trial PIS and completing an electronic trial consent form (trial consent form). Participants who provide consent are automatically emailed a copy of the trial PIS, privacy policy and completed trial consent form.

Individuals who are not suitable at pre-screen or screening (e.g., outside of age range, below threshold on RNT) will automatically be directed to a webpage explaining why they are not suitable for the trial. Those reporting mental health difficulties will be automatically guided to webpages providing information, guidance including to consult with their general practitioner (or equivalent), and weblinks and telephone numbers for help and support, including contact details for the trial team.

### Baseline and follow-up assessments

The baseline assessment will take place after electronic informed consent to participate in the trial is provided and consists of web-based self-report measures to assess current and lifetime history of depression, current wellbeing, symptoms of anxiety and depression, social and work functioning, use of services and treatment received, academic grades, resilience, and stressful events, and levels of RNT (see outcome measures and Tables 1 and 2). The Lifetime Depression Assessment Self-report questionnaire (LIDAS) [42] will be used to assess lifetime major depression (MDD) diagnosis according to DSM criteria and is largely based on the widely used Composite International Diagnostic Interview (CIDI). It has been proven to be effective for determining history of depression through self-report in an online digital format, matching the needs for the current study [42]. It consists of a conditional sequence of pre-programmed questions assessing all the diagnostic criteria for depression, with logic cut-outs so that subsequent questions are determined by prior questions, keeping the assessment brief.

**Table 1:**
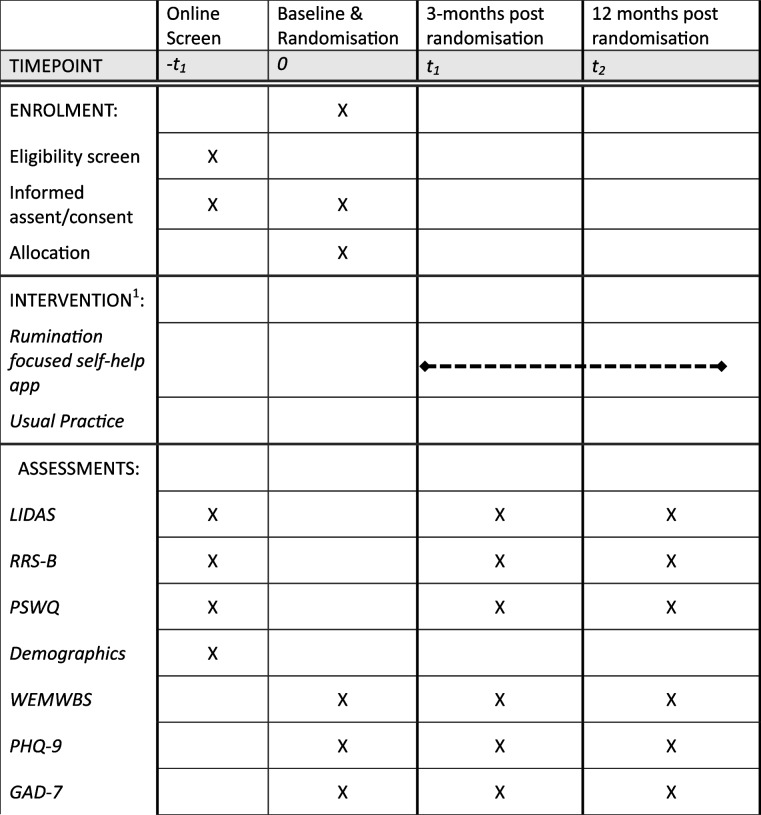
Schedule of enrolment, interventions and assessments

**Table 2:** Measurements and endpoints

			**Follow-up (months)**	
**Web Assessment**		**Baseline**	**3**	**12**
Pre-screening	date of birth, self-reported mental health	✓		
Informed Consent		✓		
Socio-demographics	Age, sex, employment status, ethnicity, historic mental health problems	✓		
LIDAS	Incidence of current and past major depressive episode (primary outcome)	✓	✓	✓
Rumination	RRS-Brooding questionnaire; secondary outcome	✓	✓	✓
Worry	PSWQ questionnaire; secondary outcome	✓	✓	✓
Wellbeing	WEMWBS questionnaire; secondary outcome	✓	✓	✓
Depression	PHQ-9 questionnaire; secondary outcome	✓	✓	✓
Anxiety	GAD-7 questionnaire; secondary outcome	✓	✓	✓
Resilience	BRS questionnaire; secondary outcome	✓	✓	✓
Perceived Stress	PSS-4 questionnaire; secondary outcome	✓	✓	✓
Stressful Events	ASQ questionnaire; secondary outcome	✓	✓	✓
Use of mental health services and resources			✓	✓
Academic Outcomes		✓	✓	✓

Once consented participants have completed the baseline assessment, they will be randomised automatically by an independent computer system. Randomised participants will be informed of their randomisation allocation and signed up to use the relevant variant of the app via clinically trained members of the research team. The participant’s University email address is used for app set-up.

**Fig. 1:**
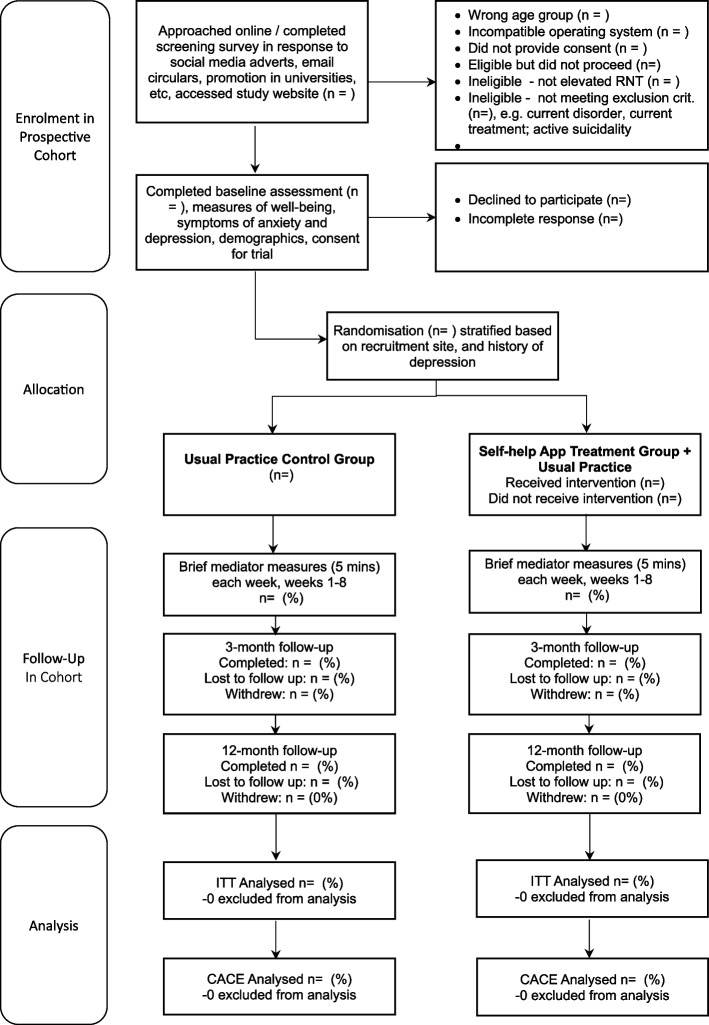
CONSORT flow diagram

All participants will be followed up electronically at 3 months and 12 months post-randomisation. At each follow-up point, participants will be automatically sent emails and texts with links to enter their data into the EDC. Each assessment point will involve an automated weekly follow- up by email and then text and telephone follow-ups to participants who haven’t yet completed the EDC assessment at 3 months and 12 months. Tables 1 and 2 give an overview of all measurements. Figure 1 gives an overview of trial flow. Project researchers will be blind to treatment allocation but will be available to participants to answer queries about the trial or the assessments. Participants will receive honorariums for the completion of each of the brief mediator assessments occurring each week for 8 weeks post-randomisation, 3-month and 12-month follow-up assessments.

### Randomisation, intervention delivery and masking

Participants will be randomised (in a 1:1 ratio) to the two intervention arms. Randomisation will be conducted automatically by means of a custom-built secure web service created and managed by the Exeter Clinical Trials Unit (ExeCTU), which interfaces with the trial database, and which will be independent of the trial researchers. To promote balance across key participant characteristics across intervention arms, randomisation will be stratified according to recruitment site (each of the six funded partner universities plus an “other” university category for students recruited elsewhere), and history of depression (no history vs past history of depression – given that this is a strong predictor of future depression and was used previously) [19]. Stratification will be used (on these two variables) and blocking will be used with minimum block size of 4 and maximum block size of 6, occurring at random to maintain concealment.

All the online recruitment and randomisation will be automated and independent of trial researchers. The Exeter CTU EDC system will contact an unblinded team member (administrator/therapist) indicating when an individual [by study ID] has been randomized to the active intervention. This team member will then access the relevant details in the EXCTU database and manually set up the participant in the mobile app intervention using administrator rights via the treatment platform dashboard. An email will also go to the participant indicating the condition to which they are randomised and informing them what to expect. The relevant unblinded team member will also monitor if the participant has accessed the intervention and check if any difficulties and encourage to sign-up.

All assessments will be routinely collected online using the EDC following automated reminders, without the involvement of researchers. Site researchers will be blind to treatment allocation. Site researchers will prompt all participants to complete follow ups by phone and text if they do not respond to the automated email reminders. Any unblinding in contact with a site researcher would be logged as a protocol deviation and only a researcher that remained blind will be able to prompt future follow-up from that participant.

### Interventions

#### Usual practice control

This control is what usual practice the participants may receive during the course of the 12-month follow-up. Usual practice, as received by the university student outside of the trial, may include no provision of intervention, local provision of intervention, support from their GP/family doctor, local health services or youth services, or provision of intervention within their university (e.g., well-being service; support and welfare staff). The nature of usual practice will be monitored and assessed by questionnaires at each follow-up assessment, determining what treatment and services participants have received since the last assessment.

#### Unguided RNT-focused self-help mobile app (experimental intervention group)

The self-help app includes self-monitoring, psychoeducation, and active self-help exercises based on RNT-specific strategies from an evidence-based rumination-focused CBT intervention [35]. Core elements of the intervention are designed to break the ruminative habit and enable users to shift towards a more helpful processing style. This involves coaching participants to spot warning signs for rumination and worry, and then to plan alternative strategies. These include being more active, slowing things down, breaking tasks down, opposite action, relaxation, concrete thinking, becoming absorbed, self-compassion, and assertiveness. Participants are prompted to practice alternative strategies in response to their warning signs. The user interface includes text, pictures, audio-recordings, vignettes, animations, audio-exercises, quizzes, and questionnaires with tailored automated feedback. The app is entirely automated (i.e., self-guided) and designed for use on both iOS and Android phones. The app is accessed for free via each participant’s smartphone app store and is set up in the Minddistrict delivery platform. The app is structured in a logical order with participants needing to complete each module/lesson before proceeding to the next lesson. Each lesson can be completed within 5-30 mins and followed a structure of psychoeducation, student stories, experiential exercises, strategies to practice, making plans, and a quiz to test knowledge (see Table 3 for further detail).

**Table 3:** Detailed description of internet RNT-focused mobile app intervention

**Module Name**	**Description of contents**
**Building Confidence and Reducing Worry** (introductory module) App is programmed so that all users receive this first – they can then choose in which order they want to complete the subsequent modules – all subsequent modules introduce new strategies to add to the changing habit framework introduced in first module. Participants work through the module in order with various points where they choose which and how many examples or strategies they read, enabling further personalisation.	**Chapter 1: Introduction to therapy**, student stories (introducing 4 diverse students and their backgrounds and difficulties – Rory, Li Wei, Amira, Alex), psychoeducation on common problems linked to RNT (perfectionism, procrastination, imposter syndrome, academic stress, fear of missing out, bullying and harrassment, loneliness, adjusting to university), introduction to action plan, with example, activation of My Goal and Action Plan features (which user can access whenever they want in app to créate plans and which enables user to set a goal and make a plan to reach the goal); psychodeducation on worry and rumination as a habit including animated video; student stories of experiences of overthinking; quiz to check understanding **Chapter 2: Spotting Triggers** – psychoeducation on importance of spotting triggers to worry and rumination, including animation; my warning signs quiz that reviews range of different warning signs (situations, physical reactions, places and times, actions, thoughts) for RNT and gives contingent feedback on what actions and modules may be helpful; quiz to check understanding; Mood Tracker tool activated that enables users to record at any time new triggers for RNT and stress **Chapter 3: Removing and interrupting triggers** – psychoeducation on value of removing triggers to RNT to change habit, including animation; choice of tools to manage triggers to review including detailed steps, tips, and student examples for (a) sleep hygiene (including regular routine, stimulus control),(b) changing routine and environment (e.g., taking a break), (c) slowing things down; (d) breaking tasks down into smaller steps; (e) being more active (including SMART plans); (f) worry postponement and worry periods; quiz to check understanding; activation of Worry Diary that participant can access to and add a new entry whenever they want which helps them to note a worry and then to return to later at a scheduled worry time. **Chapter 4: Learning new helpful habits** – psychoeducation on how to learn new habits including animated video; introduction of IF-THEN plans (implementation intentions to plan an alternative to RNT when notice warning signs for RNT, e.g., If I notice I am getting tense, then I will listen to a relaxation exercise) as useful way to plan new responses to counter RNT, with student example; introduction of different alternative helpful responses to RNT; introduces Opposite action as a useful alternative with animated video; opposite action tool is interactive tool that suggests alternative actions, facial expressions and body language to try in response to anger, anxiety, shame, sadness, boredom or tiredness; IF-THEN diary is activated, in which participant can add a new IF-THEN plan and reminder whenever they want; quiz to check understanding
**Helpful Thinking to Build Confidence and Reduce Worry**	Psychoeducation on how different processing styles (abstract Why? vs concrete-specific How?) influence effects of RNT, including animated video; behavioural experiment including audio-recorded exercises to compare different effects of abstract versus concrete thinking; steps and tips to think more concretely and be more specific; activation of Concrete Thinking tool (audio-exercise to practise being more specific); explanation of how changing thinking is a potential alternative to RNT in the IF-THEN plan and making a new IF-THEN plan; activation of Be Specific diary that participant can open and complete a new entry whenever they want, with prompts to aid them in thinking in a more specific way; quiz to check understanding
**Relax and Refocus to Build Confidence and Reduce Worry**	Psychoeducation on relaxation and how it might be useful for RNT; choice of 3 different relaxation exercises to practice, each with audio-recording (Slow Breathing; Body Scan, Progressive Muscle relaxation); practice of exercise with review of effect; explanation of how relaxation is a potential alternative to RNT in the IF-THEN plan and making a new IF-THEN plan
**Being Absorbed to Build Confidence and Reduce Worry**	psychoeducation on how being absorbed in an activity and connecting with experience act counter to RNT; behavioural experiment using audio-recorded exercises to compare experience of times when absorbed versus time not absorbed; explanation of how absorption is a potential alternative to RNT in the IF-THEN plan and making a new IF-THEN plan; access to absorption audio-recorded exercise as tool; making plans to increase absorbing activities, using My Activity Plan feature; student example; quiz to check understanding
**Being Kind to Yourself to Build Confidence and Reduce Worry**	psychoeducation on how being self-compassion and kindness act counter to RNT including animated video; behavioural experiment using audio-recorded exercises to compare experience of times when being kind versus time not being kind; explanation of how self-compassion is a potential alternative to RNT in the IF-THEN plan and making a new IF-THEN plan; access to self-compassion listening exercise; choice of tips on how to talk to oneself in a kinder way; making plans to increase self-compassionate activities, using My Activity Plan feature; student example; activation of Be Kind diary that participant can open and complete a new entry whenever they want, with prompts to aid them in thinking about a situation in a more supportive and tolerant way; quiz to check understanding
**Developing Assertiveness to Build Confidence and Reduce Worry**	Psychoeducation on what assertiveness is and how it can be helpful response to reduce RNT; choice of student stories as examples; choice of communication styles to read about; exercise about how to be more assertive providing tips, with student examples; choice of different tips about being assertive to read; quiz to check understanding
**Building Confidence and Reducing Worry into the Future** (this module is designed to be the last module, helping participants with ongoing practice and relapse prevention plans)	Review of what participant has learnt through the modules so far (warning signs, useful tools); encouraged to check-in regularly; activation of Check In diary that participant can open and complete a new entry whenever they want, with prompts to aid them in thinking about whether they are facing any warning signs and to review what helpful actions they can take; choice of student stories for all four students highlighting their journey and progress and what they found helpful; goal setting with choice to identify risks and make goals and future plans to stay well over 3-month, 6-month, and/or 12-month periods; questionnaire on experience of therapy; congratulations on completing course.

The content will focus on providing psychoeducation, tips, advice, strategies, and reflective exercises and learning tests relevant to reducing worry and rumination and building confidence. It has been adapted to be usable in a mobile app format and has been tailored through two iterations (one on paper with illustrative examples for a student focus group, one with n=6 students working through the app and providing feedback) to reflect student concerns, including specific sections on student issues and a series of diverse student vignettes illustrating different concerns and strategies through the app. The vignettes are designed to follow different student stories to illustrate different aspects of the intervention and different experiences of worry and rumination. The adaptation includes organising all information into “bite-sized” information that can be displayed on a mobile phone screen without the need to scroll down, typically with one key message per “page”, and with users then swiping forwards to the next “page” to make it user-friendly.

There is a “favourite” feature so that any exercise or video or information could be stored and accessed whenever the user wanted from their dashboard. The app also includes interactive features that the user can access whenever they want and as many times as they want, including features to complete diaries, set goals, and make plans.

#### Intervention adherence

The use of the app will be assessed and recorded including number of times the app is used, for how long, and progress through the different modules. A minimum intervention dose for the app will be defined a priori, with “compliance” defined as completion of a pre-specified minimum level of usage of the unguided self-help intervention (at least 2 modules of 7 modules in treatment package). All participants in the usual practice control will be defined as receiving the minimum intervention dose.

### Outcomes

Outcomes will be assessed at baseline (pre-randomisation) and 3 months and 12 months post-randomisation.

#### Primary outcome

The primary outcome measure will be the incidence of major depression over 12 months (primary endpoint), indexed by the LIDAS [42] assessing diagnosis retrospectively across the follow-up period.

#### Secondary outcomes

Secondary outcomes include: the Patient Health Questionnaire-9 (PHQ-9), a well-validated measure of depression [43] and the Warwick -Edinburgh Mental Wellbeing Scale (WEMWBS) [44], a widely used and well-validated measure of wellbeing. The Generalized Anxiety Disorder-7 (GAD-7) questionnaire will be used to assess anxiety symptoms [45]. The Work and Social Adjustment Scale (WSAS) [46] will be used to measure functioning with respect to work/education, home management, social leisure, private leisure, and close relationships each rated from 0 not at all impaired to 8 severely impaired. A bespoke questionnaire will assess the use of university-based and non-university-based healthcare (including National Health Service) services, resources, and support. Another bespoke questionnaire will ask participants to report on the actual academic marks and their academic targets for the previous year and whether they were satisfied with their academic progress. The Brief Resilience Scale is a brief and reliable means to assess the ability to bounce back from stress [47]. The Perceived Stress Scale-4 is a four-item measure of individuals perceived levels of stress and ability to cope [48]. The Adverse Events Questionnaire is a brief measure designed to assess stressful events in young people, which is proven to predict subsequent depression [49]. It consists of 3 questions asking about relevant adverse experiences (bad experiences concerning academic study; bad experiences concerning relationships; other bad experiences) rated from 0 “No”, 1 “yes, happened once”, 2 “yes happened twice”, 3 “yes, happened more than twice”. A fourth item asks about minor problems or hassles ranging from 0 “minor problems” to 4 “large number of minor problems and hassles”.

Participants will be asked to complete brief measures each week for eight weeks after randomisation to provide potential mediators of change: a two-item measure of depression (PHQ-2) [50]; a two-item measure of anxiety (GAD-2) [51]; a single item measure of stress over the last week (scored 0 none to 4 very severe); six items from the Cognitive and Behavioural Response to Stress Scale [52] assessing the frequency and usefulness of use of cognitive reinterpretation, behavioural activation, and relaxation or meditation strategies; 3-item adaptations of the automaticity component of the Self-Report Habit Index [53] focused on assessing the extent to which worry and problem-solving respectively are each a habit (e.g., “something that I do automatically" “I do without thinking”) over the past seven days; 2-items from the Self-Compassion Scale-short-form [54]; 2 items from the RRS-Brooding 5-item questionnaire [40]; a one-item measure assessing self-efficacy (“In the last seven days I feel better prepared to handle situations I could not handle before” rated using a seven‐point Likert scale ranging from −3 (“absolutely not true), 0 (“neither nor”) to +3 (“absolutely true”) and a one-item measuring problem clarification (“In the last seven days, I understand myself and my problems better” rated using a seven‐point Likert scale ranging from −3 (“absolutely not true), 0 (“neither nor”) to +3 (“absolutely true”).

The following descriptive variables will be assessed only at baseline: age, gender, sexuality, year of study, course of study, family’s occupational status, country of birth.

### Sample size

The sample size was calculated using the primary outcome based on a minimum clinically important difference (MCID) at primary endpoint allowing for power at 0.80 and a two sided alpha at 0.05. The primary outcome is incidence of major depression across the 12 months follow-up. Assuming that the MCID is an absolute risk reduction in incidence of major depressive episodes of 10% based on previously published consensus [55] and that incidence of major depressive episodes will be 25% in the usual practice arm, paralleling previous studies in this high-risk sample [18, 19], and assuming 20% follow-up attrition, then conservatively we require n=324 per arm, giving a total sample required of 648.

### Statistical analysis plan

The primary analyses will be intention-to-treat (ITT) analyses [56] (i.e. all participants will be included in the analyses according to their randomised allocation) and based on complete case outcome data. The primary inferential analyses will compare across trial arms for the primary and continuous secondary outcomes at 12-months follow up using multilevel mixed-effect models, which enable us to examine nested hierarchies in the data (individual, intervention, university), across time (examining pre-to-post change), to capture dependencies in the data, to investigate individual trajectories (random intercepts, random slopes), and which have less restrictive assumptions re missing data.

Secondary analysis will be undertaken using both Complier Average Causal Effect models and multiple imputation:

- Complier Average Causal Effect (CACE) analysis [57, 58] to provide an estimate of a treatment effect accounting for pre-specified per protocol adherence and compliance with the treatment, whilst retaining the benefits of randomisation.
- Multiple imputation following the examination of the pattern of missing outcomes to impute primary and secondary continuous outcomes. Imputation models will be informed by treatment arm, baseline scores, other covariates to be included in the model, and other baseline characteristics found to predict outcome or propensity for missingness (logistic regression models will be used to investigate the associations between baseline characteristics and missingness).

Secondary analyses will be undertaken including:

- Models will be estimated for the primary and secondary outcomes at 3- and 12-month follow-up and will include the covariates adjusted for in the linear regression models.
- Cox regression models will be undertaken to investigate between group differences in the time until a major depressive episode.
- Mediation analyses will be undertaken to gain insight into mechanisms that could explain the potential effect of the interventions on primary outcomes. We will use modern causal inference methods using structural equation modelling or parametric regression models to assess mediation effects [59]. In addition, we will investigate potential moderation of the interventions by site and history of depression.
- The results of all models will be compared to primary analysis complete case ITT results.
- Analyses will be undertaken by a statistician blinded to group allocation and using Stata v.17.

### Organization, quality assurance and data management

Research data will be automatically collected in a pseudonymised manner through an electronic data capture system licenced/programmed by Exeter Clinical Trials Unit (CTU). In the first instance all participants will be directed to the electronic data capture system to provide their data. Participants who respond to follow-ups by telephone will have their primary outcome data entered into the system by a site researcher and this variation in data collection method will be recorded in the EDC. All data will be kept securely and confidentially and only accessed by specified researchers at the University of Exeter. The central data-management team will use de-identified backups for the monitoring of the overall progress and data quality. Ultimately, a comprehensive de-identified dataset will be produced that includes all outcome data.

### Trial status

The Trial was registered in ISRCTN, number of identification 86795807. Date of registration: 27 October 2022. Recruitment commenced in July 2023 and is ongoing.

## Discussion

In recent years, there has been evidence for increasing prevalence of poor mental health amongst university students, with high rates of anxiety and depression (1-5). These mental health problems have significant negative impact on students, including resulting in greater rates of university drop-out, lower grades and subsequent impact on future careers. Furthermore, many of the students who experience mental health difficulties do not receive treatment (6-8). Finding better ways to prevent the onset of common mental health problems such as depression is one necessary step to improve mental health in higher education. However, to date, there is limited evidence on effective approaches to preventing depression within university students (7-10).

Effective approaches to improve prevention need to be widely accessible and highly scalable so that they can be reach large numbers of young people. One potential approach to delivering a scalable intervention is through mobile apps (m-health) as the majority of young people regularly use mobile devices. The current study is an important contribution to this field by aiming to deliver a well-powered trial of a self-guided mobile app for university students.

Because this intervention is based on a smartphone app and is entirely self-help, without requiring support or therapist time, it has potential to be scaled up to be widely available as a public health intervention. If proven to be efficacious, this work may thus contribute to large-scale effective preventions for young people, i.e., massive open online interventions (MOOI) [23].

## Data Availability

Anonymised datasets arising from this trial will be made available after the primary outcomes are published to researchers and other groups via request to a data committee within the trial consortium. The results will additionally be updated on https://www.isrctn.com/ISRCTN86795807. We plan to communicate trial results through peer-reviewed open access publications and direct reports to TSC, sponsor, and participants.

## Abbreviations

AE: Adverse Event
CACE: Complier average causal effect
DMEC: Data Monitoring Ethics Committee
DSM-IV: Diagnostic and Statistical Manual of Mental Disorders, Fourth Edition
EDC: Electronic Data Capture System
ExeCTU: University of Exeter Clinical Trials Unit
GAD-7: Generalized Anxiety Disorder-7
ITT: Intention-to-treat
MDD: Major depressive disorder
MI: Multiple imputations
NIHR: National Institute for Health Research
NRES: NHS National Research Ethics Service
PHQ-9: Patient Health Questionnaire
PIS: Participant Information Sheet
RCT: randomised controlled trial
SAE: Serious Adverse Event
TSC: Trial Steering Committee

## Data availability

No datasets were generated or analysed during the current study.
